# Exploring the potential of incremental feature selection to improve genomic prediction accuracy

**DOI:** 10.1186/s12711-023-00853-8

**Published:** 2023-11-09

**Authors:** Felix Heinrich, Thomas Martin Lange, Magdalena Kircher, Faisal Ramzan, Armin Otto Schmitt, Mehmet Gültas

**Affiliations:** 1 Breeding Informatics Group, Department of Animal Sciences, Georg-August University, Margarethe von Wrangell-Weg 7, 37075 Göttingen, Germany; 2 Institute for Animal Breeding and Genetics, University of Veterinary Medicine Hannover, Bünteweg 17p, 30559 Hannover, Germany; 3 Institute of Animal and Dairy Sciences, University of Agriculture Faisalabad, Jail Road, 38000 Faisalabad, Pakistan; 4 Center for Integrated Breeding Research (CiBreed), Georg-August University, Albrecht-Thaer-Weg 3, 37075 Göttingen, Germany; 5 Faculty of Agriculture, South Westphalia University of Applied Sciences, 59494 Soest, Germany

## Abstract

**Background:**

The ever-increasing availability of high-density genomic markers in the form of single nucleotide polymorphisms (SNPs) enables genomic prediction, i.e. the inference of phenotypes based solely on genomic data, in the field of animal and plant breeding, where it has become an important tool. However, given the limited number of individuals, the abundance of variables (SNPs) can reduce the accuracy of prediction models due to overfitting or irrelevant SNPs. Feature selection can help to reduce the number of irrelevant SNPs and increase the model performance. In this study, we investigated an incremental feature selection approach based on ranking the SNPs according to the results of a genome-wide association study that we combined with random forest as a prediction model, and we applied it on several animal and plant datasets.

**Results:**

Applying our approach to different datasets yielded a wide range of outcomes, i.e. from a substantial increase in prediction accuracy in a few cases to minor improvements when only a fraction of the available SNPs were used. Compared with models using all available SNPs, our approach was able to achieve comparable performances with a considerably reduced number of SNPs in several cases. Our approach showcased state-of-the-art efficiency and performance while having a faster computation time.

**Conclusions:**

The results of our study suggest that our incremental feature selection approach has the potential to improve prediction accuracy substantially. However, this gain seems to depend on the genomic data used. Even for datasets where the number of markers is smaller than the number of individuals, feature selection may still increase the performance of the genomic prediction. Our approach is implemented in R and is available at https://github.com/FelixHeinrich/GP_with_IFS/.

**Supplementary Information:**

The online version contains supplementary material available at 10.1186/s12711-023-00853-8.

## Background

In the last two decades, new advances and technologies in the field of sequencing—commonly referred to as next-generation sequencing—have dramatically decreased the cost and time necessary for genotyping an individual [1, 2]. The resulting abundance of genomic data was the prerequisite for genomic prediction to become an important tool in the field of animal and plant breeding [3–6]. Genomic prediction is the process of predicting phenotypic values based on genomic data in the form of genetic markers. Nowadays, these markers are typically biallelic single nucleotide polymorphisms (SNPs). The predicted phenotypes can then be used as an alternative to the actual phenotyping of individuals, which can be an expensive and time-consuming process, and, thereby, speed up the process of breeding programs and increase their efficiency [4].

Since Meuwissen et al. [7] popularized the concept of genomic prediction, many different algorithms have been developed. These include among others, linear regression based approaches such as best linear unbiased prediction (BLUP) and different Bayesian algorithms (e.g. BayesA, BayesB, BayesC, BayesCπ, or Bayesian ridge regression) as well as additional machine learning models, such as support vector machines, random forest or neural networks [8, 9]. To date, no single method has proven to be the universally best approach and their performances depend highly on the dataset being studied [9, 10]. However, Howard et al. [8] suggested the use of non-parametric models if the genetic architecture of the trait is not known to be strictly additive and includes non-additive effects such as interactions between loci. Regardless of the model chosen, accurate phenotype prediction generally requires a large number of genetic markers that ideally cover all loci involved in the trait.

However, with the increasing availability of SNPs, the number of genotyped and phenotyped individuals has become the limiting factor for the development of genomic prediction models [10]. This leads to the so-called *p* ≫ *n* problem, where the number of features (*p*)—in this case SNPs—is much larger than the number of individuals (*n*), which can result in overfitted models with poor performance [11]. One way to solve this problem is to use a feature selection approach in order to reduce the number of SNPs in the dataset, which in addition would save computation time and resources [12–15]. Different feature selection approaches have been applied for genomic prediction. These range from simple filters that remove uninformative or redundant SNPs (e.g., using a variance threshold or linkage disequilibrium pruning) [16] to more complex algorithms such as Elastic Net, BayesA, least absolute shrinkage and selection operator (LASSO), gradient boosting machine (GBM), and others that are used to identify and select relevant SNPs [9, 12, 13, 17]. In their study on the prediction of human traits, Bermingham et al. [14] applied a feature selection approach that ranks the SNPs based on the strength of their association with the phenotype as determined by a genome-wide association study (GWAS). A similar approach was used by Jeong et al. [15] for their feature selection program GMStool. Both studies reported that a GWAS-based feature selection approach may improve the accuracy of genomic prediction. However, it is still unclear under which circumstances feature selection results in an actual improvement compared to using all available SNPs. To address this, we describe an incremental feature selection approach based on GWAS, similar to [14, 15], in combination with random forest as a prediction model and demonstrate its effectiveness by applying it on several animal and plant datasets. Our results suggest that the incremental feature selection approach may lead to a considerable improvement in prediction accuracy, but it depends strongly on the genotype data. Therefore, we have implemented our approach as an easy-to-use R script to enable researchers to decide for themselves if their dataset would benefit from incremental feature selection.

## Methods

### Data

We analyzed several publicly available genotype-phenotype datasets for animals and plants, some of which have already been used for genomic prediction [9, 18, 19]. In their benchmarking study on genomic prediction, Azodi et al. [9] analyzed genotypic data from six plant species, namely maize, rice, sorghum, soy, spruce, and switchgrass. For each species, they provided three phenotypes. The datasets themselves were taken from different publications and contain SNP genotypes obtained through genotyping-by-sequencing as well as SNP chips. Since population size and number of SNPs varied considerably between these species, we were able to consider the effect of feature selection under different scenarios. However, for this study, we used only five of these six datasets, i.e. the rice dataset was excluded because the missing genotypes were filled using the numeric genotype mean values, which made them unsuitable for our analysis (only 968 out of 57,542 SNPs have no imputed genotypes) [20]. Cleveland et al. [18] published a dataset containing genotypes and phenotypes for 3534 pigs, which was made available by the Genus company PIC. The genotypes were obtained using the Illumina PorcineSNP60 chip [21], and phenotypes were measured for five traits with heritability estimates ranging from 0.07 to 0.62. However, SNPs and phenotypes were anonymized, so what the real traits were, was not known. Another dataset used in this study was published by Liu et al. [19] and relates to the egg weight of 1063 Rhode Island Red chicken. The animals were genotyped using the Affymetrix Axiom® 600K Chicken Genotyping Array and egg weight was measured at seven time-points up to an age of 80 weeks. Due to missing phenotype and genotype data, the numbers of individuals and SNPs in the pig and chicken datasets varied depending on trait. Individuals and SNPs with missing values were removed. No further quality control was performed since the datasets were already filtered in their original publications. Table 1 gives a short overview of the datasets and their respective sizes.

**Table 1:** Overview of the datasets used in our study

Species	#Individuals	#SNPs	Phenotypes (abbreviation)
Maize	391	244,781	Flowering time (FT)
			Height (HT)
			Yield (YLD)
Sorghum	451	56,299	Height (HT)
			Grain moisture (MO)
			Yield (YLD)
Soy	5014	4234	Height (HT)
			Time to R8 developmental stage (R8)
			Yield (YLD)
Spruce	1722	6930	Diameter at breast height (DBH)
			Wood density (DE)
			Height (HT)
Switchgrass	514	217,150	Anthesis date (AN)
			Height (HT)
			Standability (ST)
Pig	2804	33,861	Trait 1 (T1)
	2715	33,861	Trait 2 (T2)
	3141	33,861	Trait 3 (T3)
	3152	34,468	Trait 4 (T4)
	3184	34,464	Trait 5 (T5)
Chicken	1052	294,705	First egg weight (EWAFE)
	1063	294,705	Egg weight at 28 weeks of age (EW28)
	1063	294,705	Egg weight at 36 weeks of age (EW36)
	1027	294,705	Egg weight at 56 weeks of age (EW56)
	960	294,705	Egg weight at 66 weeks of age (EW66)
	847	294,705	Egg weight at 72 weeks of age (EW72)
	852	294,705	Egg weight at 80 weeks of age (EW80)

The last column contains the full name of the phenotype as well as its abbreviation that is used otherwise

### Methodology

The incremental feature selection (IFS) approach that we used is analogous to the methods presented by Bermingham et al. [14] and Jeong et al. [15]. In order to select an optimal number of SNPs, we first performed a GWAS on the training data (80% of the individuals) by using the PLINK software (version 1.90) [22]. Based on the reported *p*-values, we ranked the SNPs according to the strength of their association with the phenotype. With the ranking in place, we began by training a model using only the top-ranked SNP as input. Subsequently, we added new markers in a stepwise manner to the input dataset and trained models with those. This stepwise incrementation was continued until a trained model that used all SNPs was obtained. As the number of features increases, the training becomes more time-consuming. To speed up the process, we increased the step size after certain intervals (e.g. step size of 1 until 100, 5 until 500, 10 until 1000, …).

To assess the impact of the IFS approach, we used a random forest (RF) algorithm for genomic prediction, although other methods for genomic prediction could also be applied. RF is a non-parametric algorithm, which works by averaging the prediction results of multiple independently trained regression trees [23]. Compared to other additive or linear approaches, RF has the advantage of being able to capture interaction effects between SNPs as well as dominance effects [15, 24]. Furthermore, RF has proven to be a robust and highly predictive model for genomic prediction which can reach performances comparable to other classical approaches such as Bayesian methods [9, 10, 15, 16, 25, 26]. We used the implementation provided in the R package *ranger* with default settings (500 trees, mtry = $\sqrt{p}$ and a minimal node size of 5) [27]. Compared to other libraries, this is a highly optimized implementation that can be parallelized to increase speed.

Cross-validation was applied to measure the ability of the trained models to predict unobserved phenotypes. Following Azodi et al. [9], we split the individuals into five separate folds, thus the training data consisted of 80% of the population and the model was tested on the remaining 20%. This fivefold cross-validation was repeated ten times with randomly reordered individuals. Previous studies have shown that only the training data should be used for feature selection since including the test data results in an inflation of the prediction accuracy [14, 28]. Therefore, the GWAS was repeated for each training dataset, separately.

Following previous studies [10, 17, 29–32], we measured the accuracy of the predictions using the coefficient of determination (*R*^*2*^), which can be interpreted as the proportion of the variance in the dependent variable that can be explained by the genotypes [33]. For each repetition of the cross-validation, we calculated the *R*^*2*^ value between the predicted and the observed phenotypes for all folds. Based on these values, we reported the average *R*^*2*^ value as well as their standard error for each model. Although Pearson’s correlation coefficient (*r*) is the most widely used measure in genomic prediction, it does not necessarily mean that a high value of *r* reflects the accurate prediction of the true phenotype values. Furthermore, a repetition of our analysis using *r* as accuracy measure demonstrated a correlation of nearly 1 between the two measures on the test results. Please note that *R*^*2*^ is equal to the square of *r* only in linear regression with no constraints [31].

To obtain a robust trend curve for the performance of the prediction model depending on the number of SNPs, we applied Friedman’s super smoother [34] on the *R*^*2*^ values. Based on these smoothed values, we determined the maximum *R*^*2*^ value to find the optimal number of SNPs.

In order to evaluate the performance of our IFS approach on data not included in selecting the SNPs and training the model, first we randomly split each dataset into two parts, with an 80%/20% ratio. Let Φ be the dataset containing 80% of the data and Ψ the remaining dataset. Subsequently, we applied the IFS approach on Φ to determine the optimal number of SNPs. During IFS, this dataset was further subdivided using the aforementioned cross-validation approach. Then, a random forest model was trained on the Φ dataset using this optimal number of SNPs. Finally, we employed the trained model to predict the phenotypes of the Ψ dataset and calculated the *R*^*2*^ value between predicted and true phenotypes.

We implemented our IFS approach for genomic prediction as an R script that is available from https://github.com/FelixHeinrich/GP_with_IFS allowing for easy use. As mentioned in the previous sections, it requires the software PLINK to be installed as well as the R libraries *ranger*, *data.table* [35] and *ggplot2* [36]. Genotype and phenotype data need to be given in the form of PLINK’s binary formats. Additional parameters such as the number of threads, the step sizes for feature selection, or the numbers of folds and repetitions for cross-validation can be easily modified by the user in the script.

## Results

**Fig. 1:**
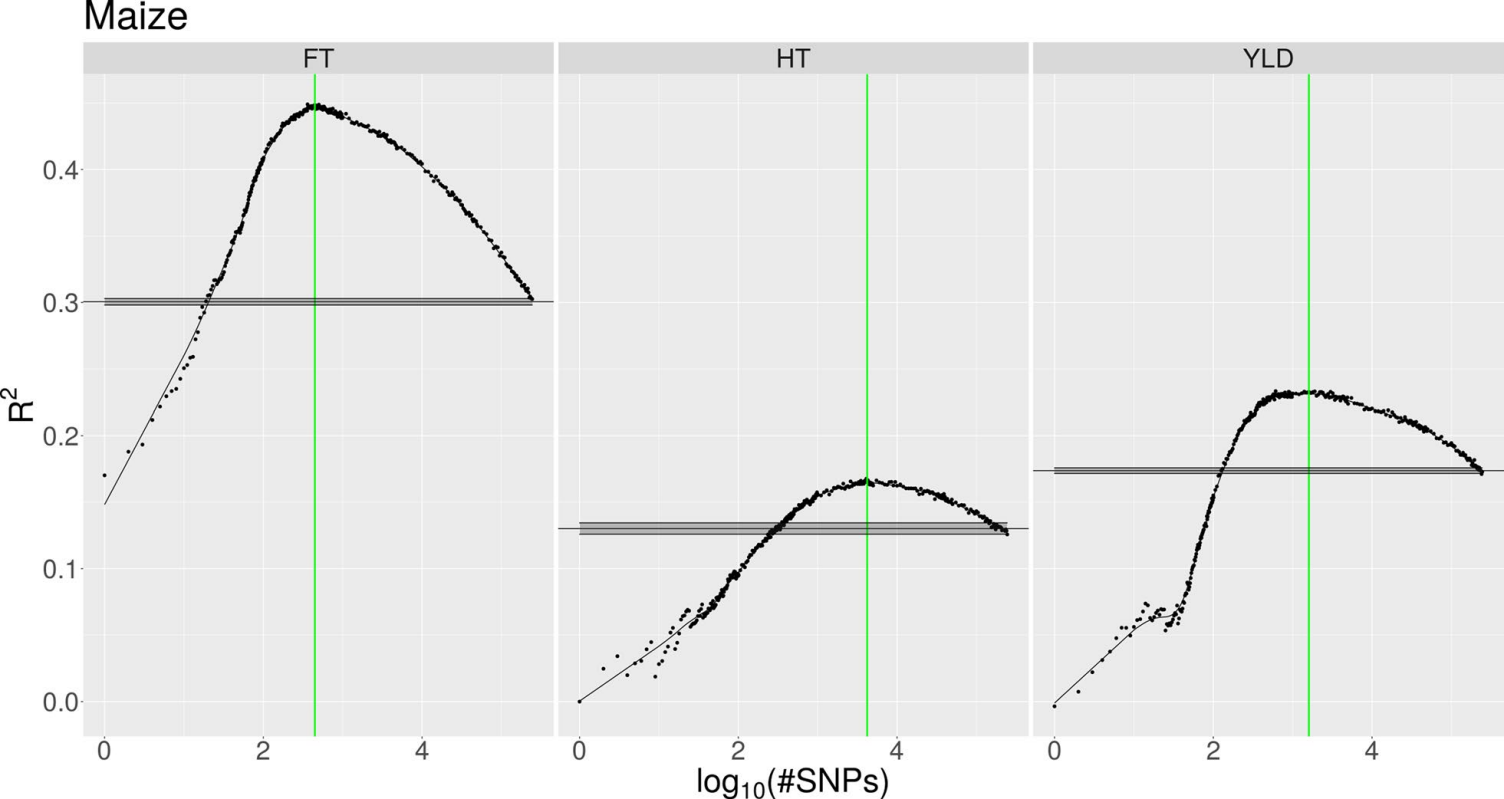
Prediction accuracy of maize phenotypes. Prediction accuracy (measured as mean *R*^*2*^) of maize phenotypes as a function of the number of SNPs used for the model (presented as logarithmic values) on the Φ data. The trend estimate, represented by the solid black curve, is obtained through smoothing. The maximum accuracy is indicated by the vertical green line. Mean performance of the model when trained on all SNPs is represented by the horizontal black line, with the shaded interval around it indicating the standard error of the mean of 10 cross-validation repetitions. The prediction accuracy of all three traits could be strongly increased using IFS with improvements ranging from 0.035 to 0.147

**Fig. 2:**
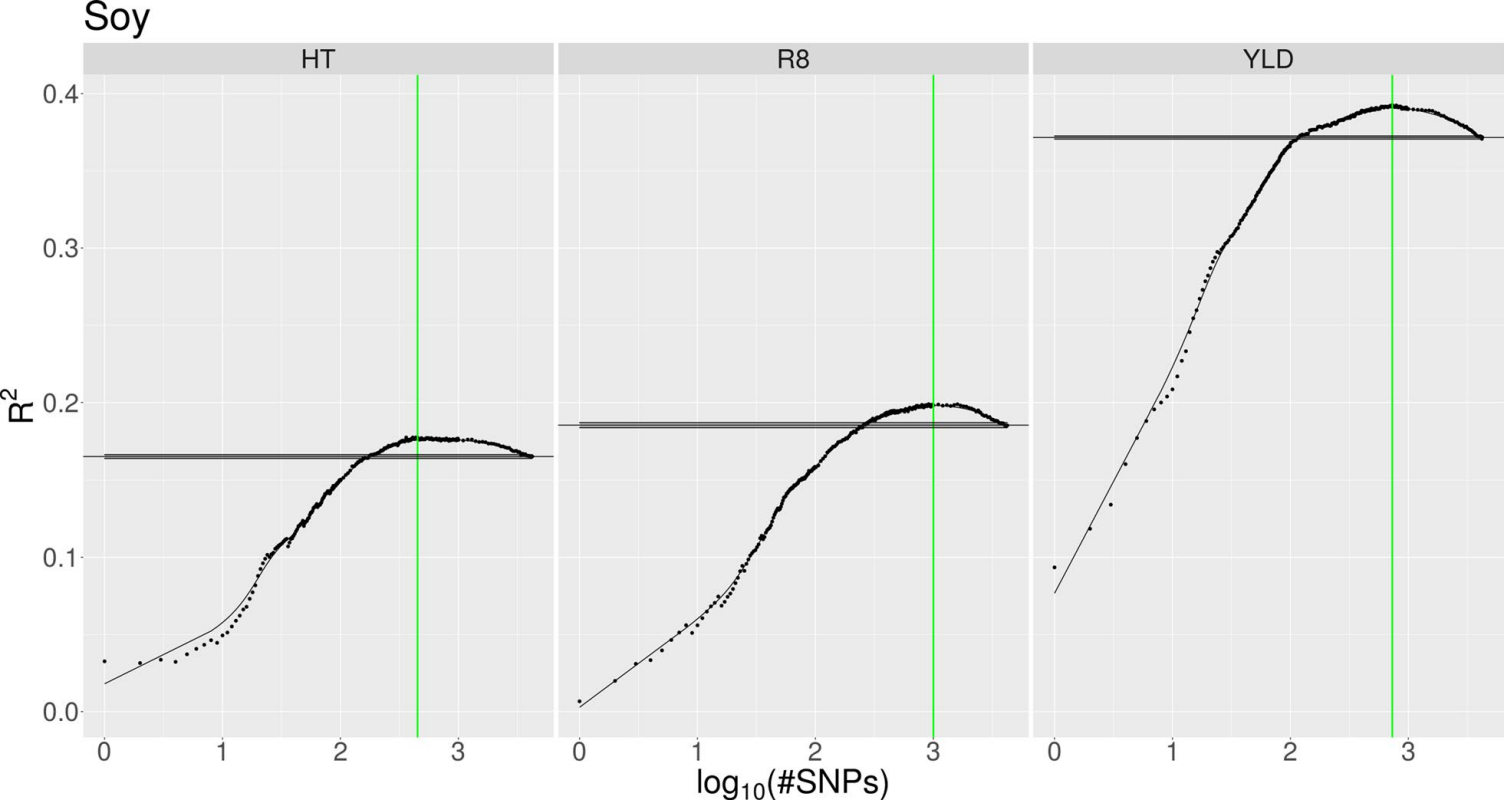
Prediction accuracy of soy phenotypes. Prediction accuracy (measured as mean *R*^*2*^) of soy phenotypes as a function of the number of SNPs used for the model (presented as logarithmic values) on the Φ data. The trend estimate, represented by the solid black curve, is obtained through smoothing. The maximum accuracy is indicated by the vertical green line. Mean performance of the model when trained on all SNPs is represented by the horizontal black line, with the shaded interval around it indicating the standard error of the mean of 10 cross-validation repetitions. The prediction accuracy of all three traits could be increased by up to 0.02 using IFS

**Fig. 3:**
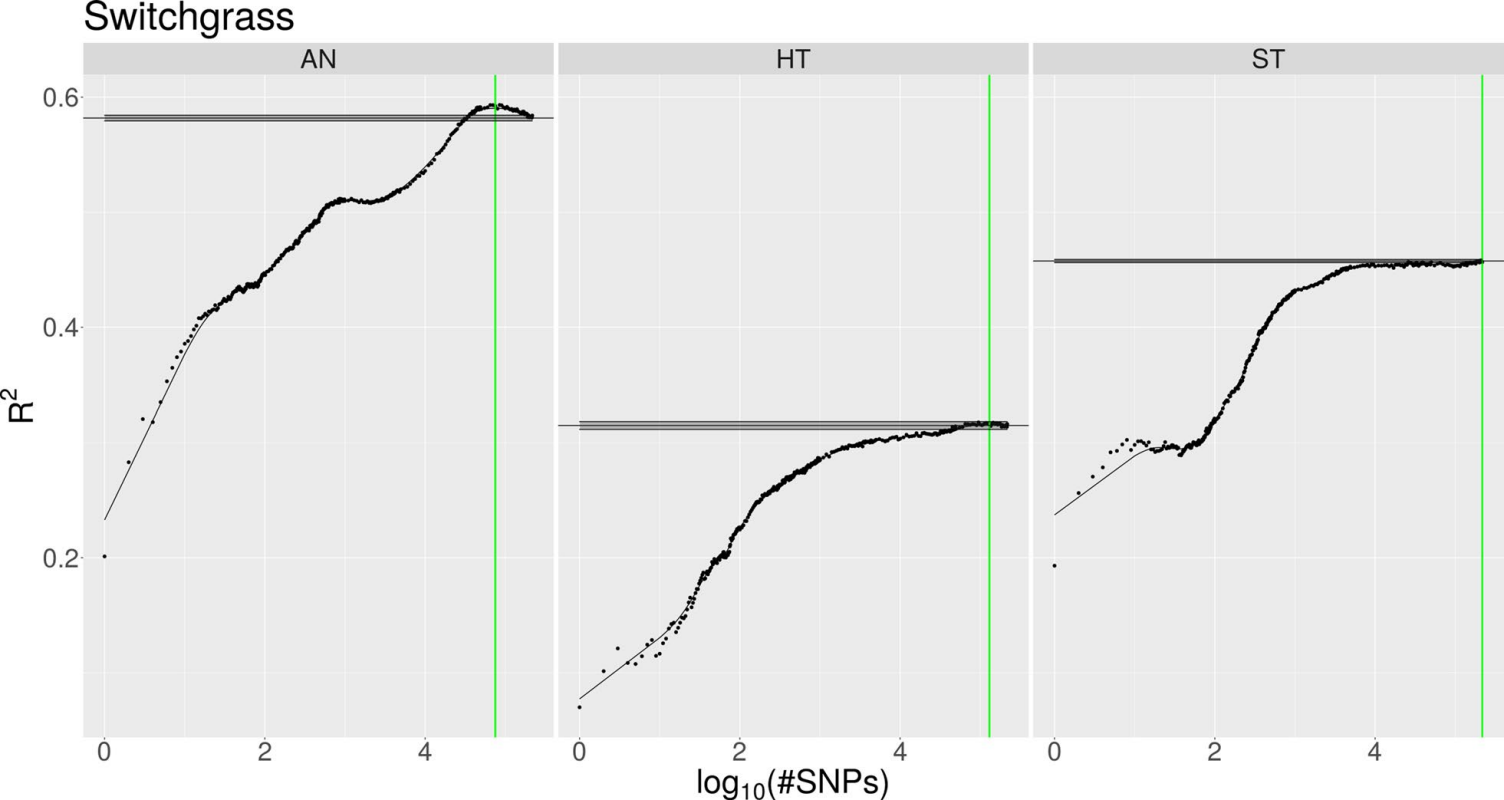
Prediction accuracy of switchgrass phenotypes. Prediction accuracy (measured as mean *R*^*2*^) of switchgrass phenotypes as a function of the number of SNPs used for the model (presented as logarithmic values) on the Φ data. The trend estimate, represented by the solid black curve, is obtained through smoothing. The maximum accuracy is indicated by the vertical green line. Mean performance of the model when trained on all SNPs is represented by the horizontal black line, with the shaded interval around it indicating the standard error of the mean of 10 cross-validation repetitions. Only for the trait of Anthesis date (AN) could the prediction accuracy be increased by 0.008

Both the increase in *R*^*2*^ obtained using the IFS approach and the baseline performance of the model trained using all available SNPs varied considerably between the different species and traits. Table 2 lists, for each dataset, the number of selected SNPs and the *R*^*2*^ value achieved with the Ψ data using the models trained only with the selected top SNPs as well as the models which were trained using all SNPs. Figures 1, 2, 3 and Additional file 1: Fig. S1, Additional file 2: Fig. S2, Additional file 3: Fig. S3, and Additional file 4: Fig. S4 show, for each species, the prediction accuracies of the phenotypes according to the number of SNPs used in the Φ data. Maize exhibited the strongest increase in *R*^*2*^ due to IFS (Fig. 1), i.e. for the prediction of flowering time (FT), with only the top 0.18% of all SNPs (446 out of 244,781) ranked on association strength, the model reached an *R*^*2*^ value of 0.486, while with all available SNPs it achieved a value of 0.364. In the following, we refer to this increase in *R*^*2*^ value as the advantage of the IFS approach. For the height (HT) and yield (YLD) traits, the advantage was 0.034 and 0.068, respectively, using the 1.64% and 0.65% top SNPs, respectively. The application of our method on the soy dataset produced similarly higher prediction accuracies although the gain was not as impressive as for maize. For soy, the advantage of IFS was between 0.003 and 0.012 when approximately the 10 to 23% top SNPs were used as input (Fig. 2). In contrast, for switchgrass, only the anthesis date (AN) and height (HT) traits showed an advantage with IFS, with an advantage of 0.013 observed using the 35% top SNPs for AN, and an advantage of 0.006 using the 60% top SNPs for HT (Fig. 3). Furthermore, for the standability (ST) trait, the model with all available SNPs reached the best performance on the Φ data. However, a similar level of performance could be achieved by using only ~ 10,000 SNPs instead of all 217,150 SNPs. In the case of sorghum and spruce, the IFS approach suggested to use all SNPs for one trait in sorghum and for two traits in spruce (see Additional file 1: Fig. S1 and Additional file 2: Fig. S2). For the other traits, the models trained on the selected top SNPs performed similarly or slightly less well with the Ψ data than the model trained with all SNPs. In the chicken dataset, all egg weight traits showed a similar behavior with respect to IFS (see Additional file 3: Fig. S3). By using less than the 20% top SNPs, the prediction accuracy could be increased by up to 0.027. Of particular note in this dataset, is that the prediction accuracy for all traits strongly decreased when the model was initially trained using the up to ~ 200 top SNPs, and the *R*^*2*^ value started to increase, only after that point, until it reached its maximum. Among the traits included in the pig dataset, the IFS approach yielded an advantage of 0.009 for the T5 trait, while it achieved a comparable or slightly less good performance than the model based on all SNPs for the other traits. This level of performance was achieved only when between 9% and 28% of the SNPs were used (see Additional file 4: Fig. S4). However, the T1 trait, which has a reported heritability of only 0.07 [18], could not be predicted at all and our approach wrongly suggested a model using only a single SNP.

**Table 2:** Prediction accuracy of phenotypes using selected SNPs compared to all SNPs on Ψ data

Species	Phenotype	#Selected SNPs (%)	*R*^*2*^ (selected SNPs)	*R*^*2*^ (all SNPs)
Maize	FT	446 (0.18%)	0.486	0.364
	HT	4021 (1.64%)	0.237	0.203
	YLD	1601 (0.65%)	0.346	0.278
Sorghum	HT	56,299 (100%)	–	0.287
	MO	27,001 (47.96%)	0.391	0.389
	YLD	8501 (15.10%)	0.003	0.030
Soy	HT	451 (10.65%)	0.213	0.201
	R8	1001 (23.64%)	0.206	0.196
	YLD	731 (17.27%)	0.373	0.370
Spruce	DBH	6930 (100%)	–	0.096
	DE	2401 (34.65%)	0.141	0.142
	HT	6930 (100%)	–	0.143
Switchgrass	AN	75,001 (34.54%)	0.767	0.754
	HT	130,001 (59.87%)	0.519	0.513
	ST	217,150 (100%)	–	0.512
Pig	T1	1 (0.003%)	− 0.021	0.013
	T2	9501 (28.06%)	0.190	0.192
	T3	3201 (9.45%)	0.080	0.079
	T4	8501 (24.66%)	0.112	0.111
	T5	7001 (20.31%)	0.172	0.163
Chicken	EW28	29,001 (9.84%)	0.046	0.046
	EW36	41,001 (13.91%)	0.088	0.070
	EW56	50,001 (16.97%)	0.091	0.079
	EW66	43,001 (14.59%)	0.116	0.102
	EW72	45,001 (15.27%)	0.015	− 0.012
	EW80	60,001 (20.36%)	0.060	0.055
	EWAFE	40,001 (13.57%)	− 0.010	− 0.014

Number of selected SNP markers based on Φ data and prediction accuracy (measured as *R*^*2*^) of phenotypes on Ψ data using random forest trained on the selected SNP markers as well as on all SNPs. If the IFS approach selects all SNPs, the corresponding *R*^*2*^ value is given as –, since it would be the same value as in the last column

## Discussion

Previous studies have reported that feature selection for genomic prediction can reduce the number of SNPs required to achieve the same performance as models trained with all available SNPs, or even result in a greatly increased performance [13–15, 30, 37]. The latter is assumed to be the case when the number of individuals is much smaller than the number of features used by the model. In this case, reducing the number of features may reduce overfitting and increase the models’ ability for generalization [38]. However, even if only a similar performance is reached with fewer SNPs, this can still lead to the development of new low-density SNP chips specific to certain traits, which can reduce the cost of genotyping and make genomic prediction more economically feasible in breeding programs [39]. In this paper, we present a feature selection approach based on incrementally adding SNPs that are sorted by their association strength to the phenotype as reported by a GWAS. This approach is an implementation of the methodology outlined by Bermingham et al. in their study on the prediction of human traits [14]. While the authors proposed further alternative feature selection methods that incorporate linkage disequilibrium (LD) information to remove redundant markers, their results indicated that these modifications did not improve the results of feature selection [14]. Furthermore, it has been shown that removing SNPs in LD can negatively impact prediction accuracy [40]. Therefore, we did not include LD pruning in our method. This IFS approach was combined with random forest as a prediction model, which has previously been shown to be a robust and highly predictive model for genomic prediction [10, 16, 26]. Furthermore, while random forest is able to consider interactions between SNPs [41], in high-dimensional data such interactions can be missed by the model [42, 43]. Therefore, reducing the number of SNPs through IFS can help in this regard.

A further advantage of the IFS approach is that it can be combined with most genomic prediction models. Several studies have shown that it is the choice of prediction model that has the greatest impact on the accuracy of the predictions [16] and that the best performing method can vary with the dataset [9, 10]. For simplicity and because of the advantages already mentioned, we applied random forest in our study. However, in the supplied R script it can be easily replaced by a different model.

We applied our approach to datasets for seven animal and plant species, each with multiple traits, and observed a wide range of results (Table 2) that went from a considerable improvement of the prediction accuracy of traits (e.g., in the maize dataset) to no improvement for several traits i.e. the IFS approach did not lead to a model that was better than that trained with all SNPs. In a few cases, the performance of the full model could also be achieved using a fraction of the SNPs. To find an optimal number of SNPs resulting in the best performance, we smoothed the *R*^*2*^ values and determined the maximum value among them. This approach works quite well if there is a peak in the trend curve (for example, Fig. 1 or 2). However, if there is no such peak and instead the trend curve strives to the asymptote that represents the performance of the full model (for example the ST trait in Fig. 3), the identified maximum tends to be more conservative and suggests to use more SNPs than what is actually necessary. A manual selection might further reduce the number of SNPs that are required to reach the best performance in such situations.

Of particular interest are the results for the chicken dataset. For all the traits in this dataset, the prediction accuracy started to decrease after about the first 30 SNPs and then started to increase again only after about 200 SNPs (see Additional file 3: Fig. S3). Discarding these SNPs, which initially lead to lower prediction accuracy, could improve the final model. As an example, for egg weight at 36 weeks of age (EW36), for which the minimum prediction accuracy was achieved using the top 236 SNPs, we examined how the removal of these influenced the prediction accuracy (see Additional file 5: Fig. S5). We found that the new curve followed a similar trend, but the model that did not discard these SNPs reached a higher prediction accuracy. Thus, although they have a negative effect on the prediction accuracy when they are first added to the model, these SNPs still have a positive contribution on the final model. Therefore, we decided not to discard any SNPs in our approach.

The number of individuals and markers in the datasets under study differed strongly between the species, which allowed us to consider different scenarios. The aforementioned *p* ≫ *n* problem suggests that feature selection should be more effective if the markers largely outnumber the individuals in the dataset [37]. However, this expectation is not reflected by our results. For example, on the one hand, with the soy dataset that included a comparable number of individuals and markers (5014 and 4234, respectively) we could improve the prediction accuracy by up to 0.012 using about 20% of the top SNPs. On the other hand, with the switchgrass dataset that included only 514 individuals and 217,150 markers, we could only improve the prediction accuracy for two of the three traits by 0.013 and 0.006, respectively. The prediction accuracy of the third trait, standability, could not be improved, although only about 10,000 SNPs seemed to be necessary to achieve a similar level of performance as the model trained with all available SNPs. There is no detectable relationship between the size of the dataset and the effectiveness of the IFS approach. Therefore, our recommendation is to always test for the effectiveness of feature selection even if the number of SNPs in the dataset is small. Furthermore, IFS seems to be more influenced by the genotype data than by the trait under study. The prediction accuracy curves for a specific species follow a similar trend across the different traits.

In their feature selection program GMStool, Jeong et al. [15] applied a similar GWAS-based approach. However, instead of selecting a certain number of SNPs to be used, they selected specific SNPs that contributed positively to the model performance when they were initially added. SNPs without a positive contribution were not included and SNPs were added one by one until certain stop criteria (number of consecutive rejections or reaching target correlation) were met. To assess the effectiveness of our method, we compared it with GMStool. However, due to the greater computation time required for GMStool, we applied it only to the small datasets and to one of the larger datasets. Both methods were run on a dual Intel® Xeon® Gold 6138 Processor using 60 threads. While GMStool offers multiple different genomic prediction models, we selected random forest, which allows a better comparison with our own approach, and the results are in Additional file 6: Table S1. On average, the runtime of GMStool was four times longer than that of our script. The higher computational speed of our approach is most likely due to GMStool training more models as it uses a constant step size of 1. Jeong et al. [15] further used a different parallelization strategy (parallelizing the cross-validation instead of the random forest training) that was combined with a less efficient R package for random forest (*randomForest* [44]). For all the datasets examined, GMStool selected a smaller number of SNPs for the best model than our IFS approach. However, the performance of the resulting final models on the Ψ data was comparable between the two methods. In four of the 13 datasets analyzed, the models selected by GMStool achieved higher *R*^*2*^ values, and in eight of the other datasets, our method resulted in models with higher *R*^*2*^ values than GMStool. For the last dataset, both approaches achieved the same performance. The better performance of our IFS approach may be due to GMStool discarding the SNPs that do not improve the performance when they are initially added to the model. This can prevent the inclusion of SNPs that are, by themselves, not strongly associated to the phenotype but may be associated through interactions with other SNPs. With our method, we found that removing SNPs, which initially have a negative contribution, may lead to less accurate predictions (see Additional file 5: Fig. S5). Finally, the stop criteria may also cause GMStool to end its selection before all possible SNPs are tested. Combined with its faster computation time, the improved results of our IFS method demonstrate its state-of-the-art performance.

## Conclusions

The objective of this study was to investigate the potential of incremental feature selection to improve the accuracy of genomic prediction. To this end, we have developed a framework for incremental feature selection based on ranking the SNPs according to the strength of their association with the phenotype as determined by a GWAS. In combination with random forest as prediction model, we applied this method to various datasets for plant and animal species. The results obtained ranged from substantial or minor increases in prediction accuracy to a reduction in the number of SNPs required to achieve the performance of the full model. A substantial improvement could only be obtained in a few cases. Furthermore, we show that even with a small number of SNPs, feature selection can still improve the performance of the model. Therefore, we propose that genomic prediction programs should always test whether incremental feature selection can improve the model. Our approach has been implemented as an R script and is freely available at https://github.com/FelixHeinrich/GP_with_IFS.

### Supplementary Information

**Additional file 1: Figure S1.** Prediction accuracy of sorghum phenotypes. Prediction accuracy (measured as mean *R*^*2*^) of sorghum phenotypes as a function of the number of SNPs used for the model (presented as logarithmic values) on the Φ data.

**Additional file 2: Figure S2.** Prediction accuracy of spruce phenotypes. Prediction accuracy (measured as mean *R*^*2*^) of spruce phenotypes as a function of the number of SNPs used for the model (presented as logarithmic values) on the Φ data.

**Additional file 3: Figure S3.** Prediction accuracy of chicken phenotypes. Prediction accuracy (measured as mean *R*^*2*^) of chicken phenotypes as a function of the number of SNPs used for the model (presented as logarithmic values) on the Φ data.

**Additional file 4: Figure S4.** Prediction accuracy of pig phenotypes. Prediction accuracy (measured as mean *R*^*2*^) of pig phenotypes as a function of the number of SNPs used for the model (presented as logarithmic values) on the Φ data.

**Additional file 5: Figure S5.** Prediction accuracy of the chicken EW36 phenotype. Prediction accuracy (measured as mean *R*^*2*^) of the chicken EW36 phenotype as a function of the number of SNPs used for the model (presented as logarithmic values) with and without including the top 236 SNPs.

**Additional file 6: Table S1.** Comparison of our method (IFS) and GMStool on several datasets. The results contain the necessary computation time (in minutes), the number of selected SNPs as well as the *R*^*2*^ value for the prediction of Ψ data.

## Data Availability

The implemented IFS approach and the datasets used in this study are available from https://github.com/FelixHeinrich/GP_with_IFS.
