# Supplementary figures and images for: Exploring the potential of incremental feature selection to improve genomic prediction accuracy
Source: Genet Sel Evol. 2023 Nov 9;55:78. doi: 10.1186/s12711-023-00853-8 (PMC10634161; doi:10.1186/s12711-023-00853-8)

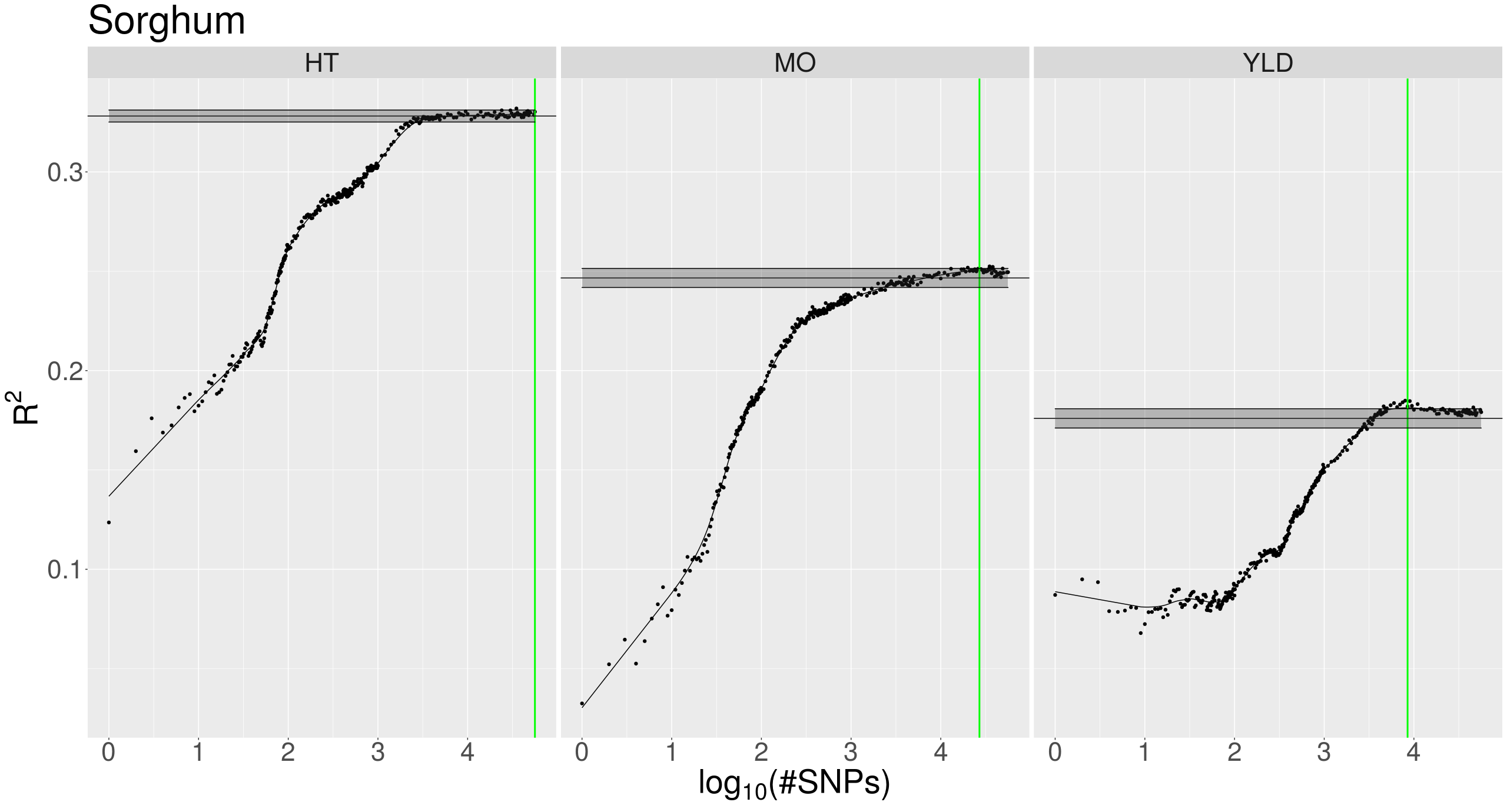

Supplement: Supplementary file 1 — Additional file 1: Figure S1. Prediction accuracy of sorghum phenotypes. Prediction accuracy (measured as mean R2) of sorghum phenotypes as a function of the number of SNPs used for the model (presented as logarithmic values) on the Φ data. [file 12711_2023_853_MOESM1_ESM.png]

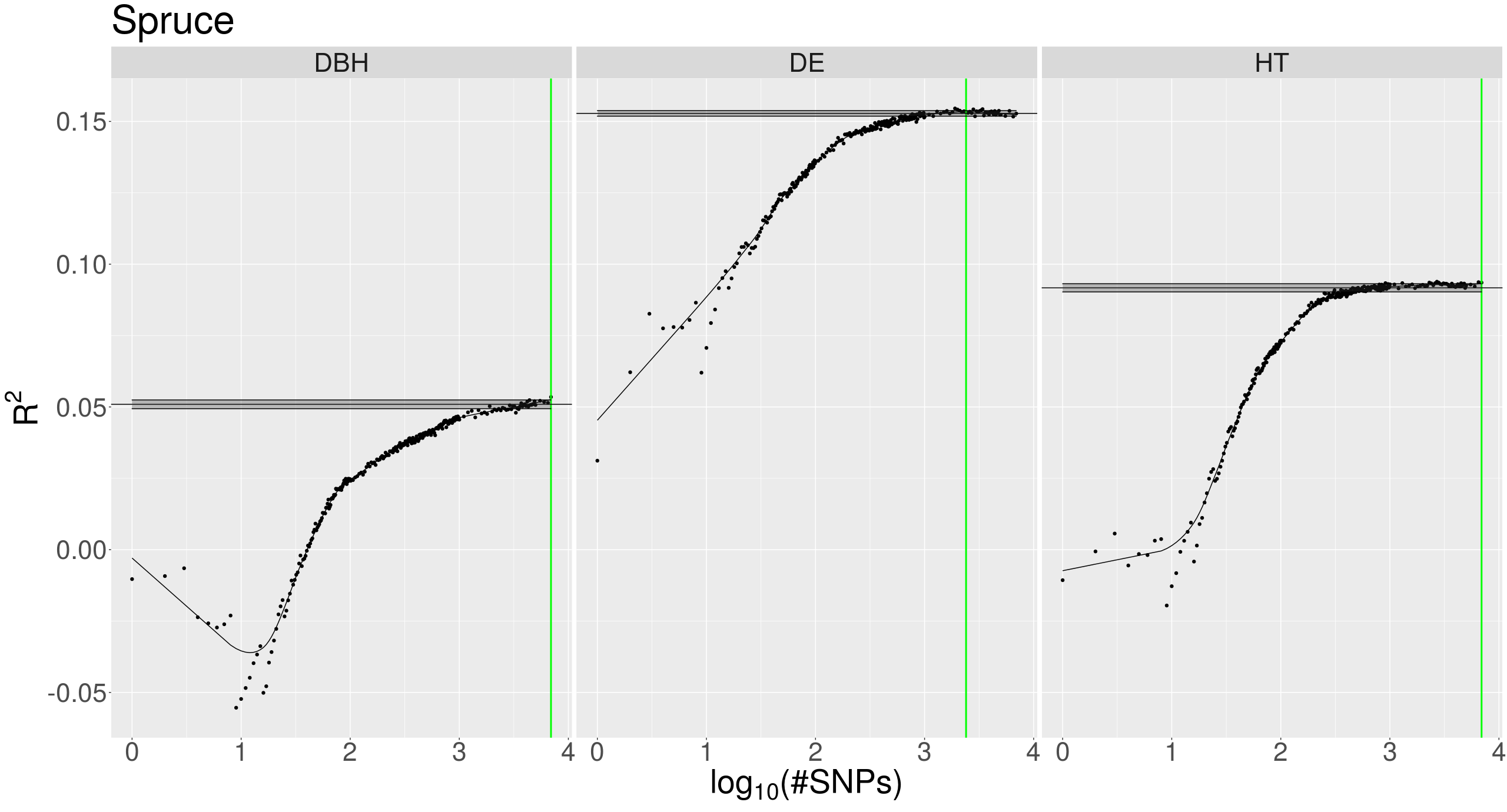

Supplement: Supplementary file 2 — Additional file 2: Figure S2. Prediction accuracy of spruce phenotypes. Prediction accuracy (measured as mean R2) of spruce phenotypes as a function of the number of SNPs used for the model (presented as logarithmic values) on the Φ data. [file 12711_2023_853_MOESM2_ESM.png]

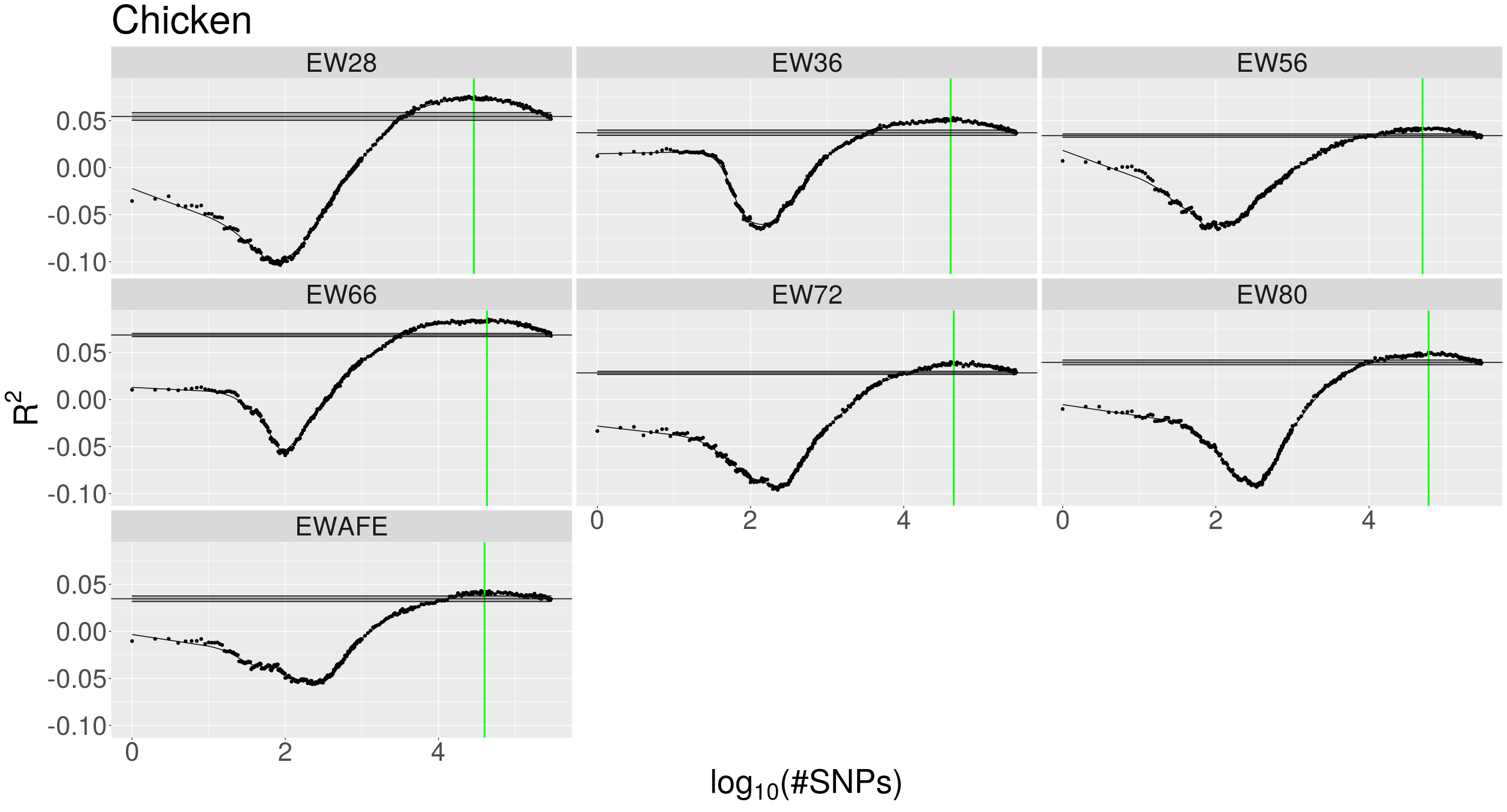

Supplement: Supplementary file 3 — Additional file 3: Figure S3. Prediction accuracy of chicken phenotypes. Prediction accuracy (measured as mean R2) of chicken phenotypes as a function of the number of SNPs used for the model (presented as logarithmic values) on the Φ data. [file 12711_2023_853_MOESM3_ESM.png]

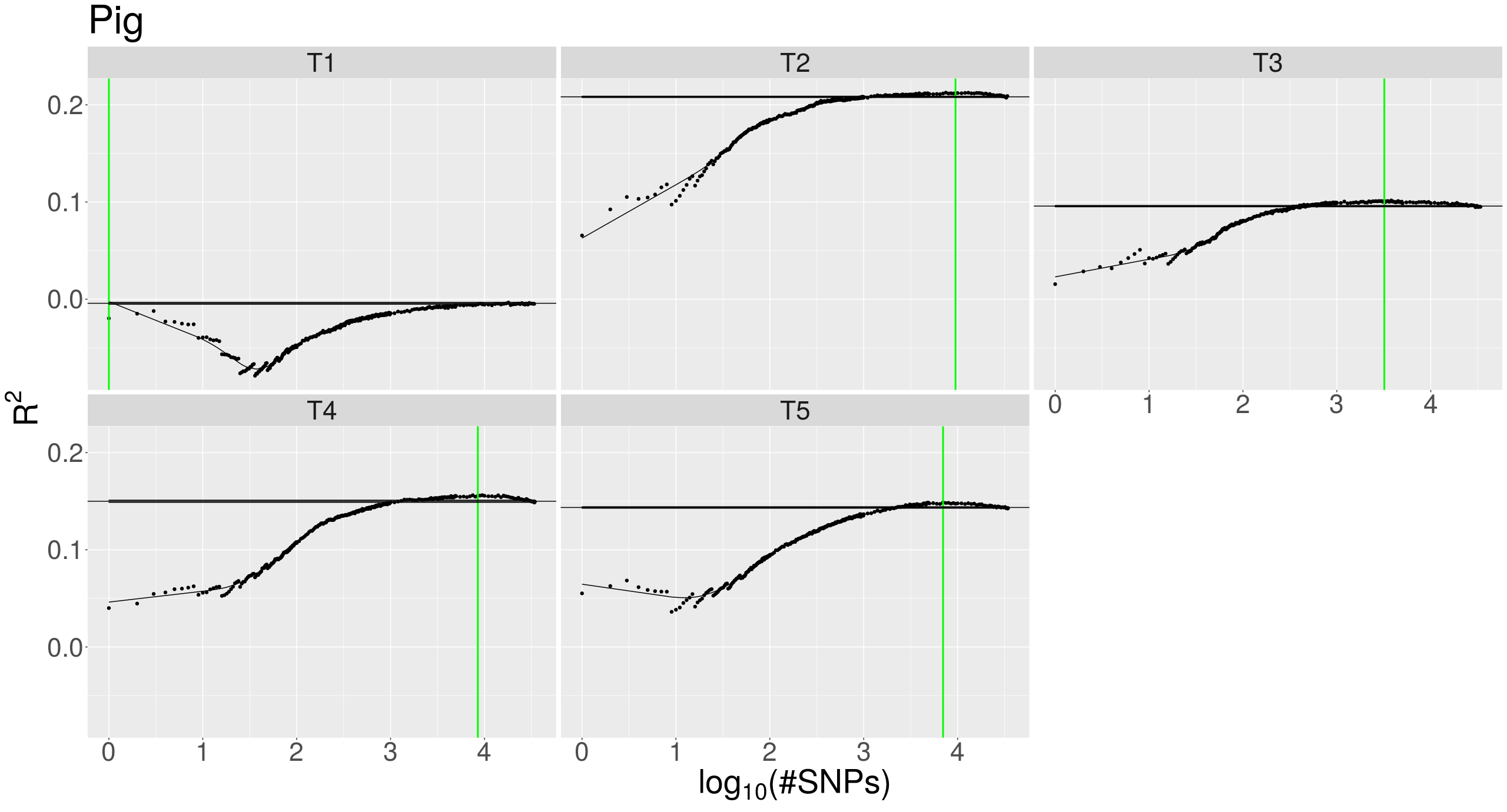

Supplement: Supplementary file 4 — Additional file 4: Figure S4. Prediction accuracy of pig phenotypes. Prediction accuracy (measured as mean R2) of pig phenotypes as a function of the number of SNPs used for the model (presented as logarithmic values) on the Φ data. [file 12711_2023_853_MOESM4_ESM.png]

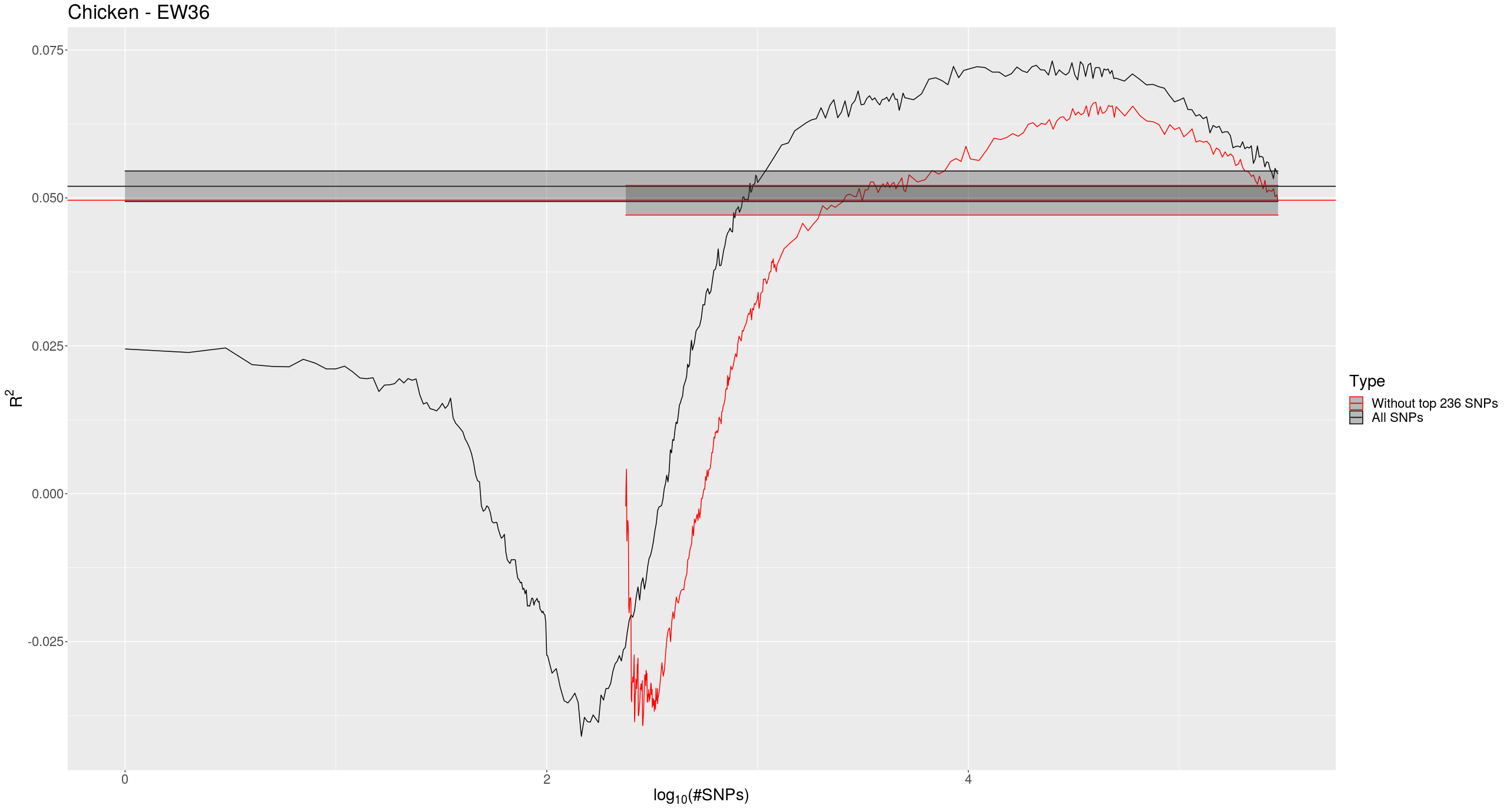

Supplement: Supplementary file 5 — Additional file 5: Figure S5. Prediction accuracy of the chicken EW36 phenotype. Prediction accuracy (measured as mean R2) of the chicken EW36 phenotype as a function of the number of SNPs used for the model (presented as logarithmic values) with and without including the top 236 SNPs. [file 12711_2023_853_MOESM5_ESM.png]
